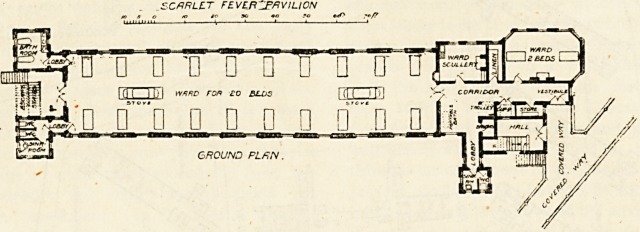# The Grove Fever Hospital at Tooting Graveney, S.W.

**Published:** 1903-05-09

**Authors:** 


### HOSPITAL ADMINISTRATION.

### CONSTRUCTION AND ECONOMICS.

### THE GROVE FEVER HOSPITAL AT TOOTING GRAVENEY, S.W.

The site of this hospital is almost quadrangular in form, and readily lends itself for hospital purposes. It is bounded northwards by Grove Road, and southwards by Blackshaw and mess-rooms. At the north-east corner is the medical education block, with post-mortem room and mortuary attached. Along the southern boundary are eight pavilions

Road. Along the eastern margin of the ground are placed the nurses' homes, and further north are the assistant nurses' quarters, the female domestics' rooms, and the sitting-rooms for the treatment of scarlet fever cases; and these pavilions are so arranged that they run due north and south, and there is no better arrangement than this, as it gives the maximum amount of sunshine and air. The pavilions are two stories high, each story consisting of one large ward for 20 beds and a small ward for two beds. At the free end of the ward are the sanitary blocks. These are properly arranged, and are cut off from the main block by cross-ventilated passages. Escape staircases are placed at this end of the block. Each bed in the ward has a window on each side of it. The end of the ward which joins the covered way contains the two-bedded room, which, we may remark, is unusually well lighted by six windows, and three of these are placed in the angles of the room, ensuring a good cross current of air. Next to this room is the linen cupboard, and between it and the large ward the scullery is conveniently placed. On the opposite side of the corridor is space for a portable bath, and a short lobby springs out of this space and leads to the nurses' lavatory, which, like the other sanitary annexes, is efficiently cut off from the block. There are also store-room, trolly-room, cupboard and broom room. The staircase to the first floor is approached from the covered way, so that there need be no unnecessary communication between the lower and upper stories. The accommodation in each block is for 44 beds—22 on each floor, giving a total of 352 beds for scarlet fever patients. The large wards are warmed by two stoves in each, placed centrally, and the small wards by open fireplaces. These pavilions, as regards their aspect, their general arrangements, their careful thinking out of details, are among the best that we have yet described in the columns of The Hospital.

The covered ways connect these various blocks with each other and with the administrative portions of the building. There is a small reception-room for scarlet fever. This room butts against the corridor, or covered way, at one end, and the other end is so placed that it has a direct communication with the road which runs from the entrance gates towards the centre of the institution. North of the covered way are six isolation blocks. Two of these contain four two-bedded wards, two contain two four-bedded wards and single-bedded rooms, and the others have six- and two-bedded rooms, so that every size of ward which can possibly be wanted has been supplied by the architect. Covered ways connect these isolation blocks, and there is another of these covered ways running east and west, to the north of which are four pavilions for diphtheria and enteric fever.

The entire accommodation for these two diseases is 112 beds The blocks are slewed round, like those for scarlet fever, to obtain the best aspect, and the wards are designed on the same principles and have the same good points as those mentioned when describing the scarlet fever pavilions. There is a receiving-room for diphtheria patients also.

A house for the steward and rooms for the male staff are provided in a block not far from the main entrance, and near this are the friends' waiting-room and the discharging-rooms. The spaces between and around the pavilions have been utilised for airing courts.

Farthest north of all is the medical superintendent's house. It is approached from the main road of the hospital and, very properly, is quite detached.

The architect is Mr. A. Hessell Tiltman, of Russell Square.

## Figures and Tables

**Figure f1:**
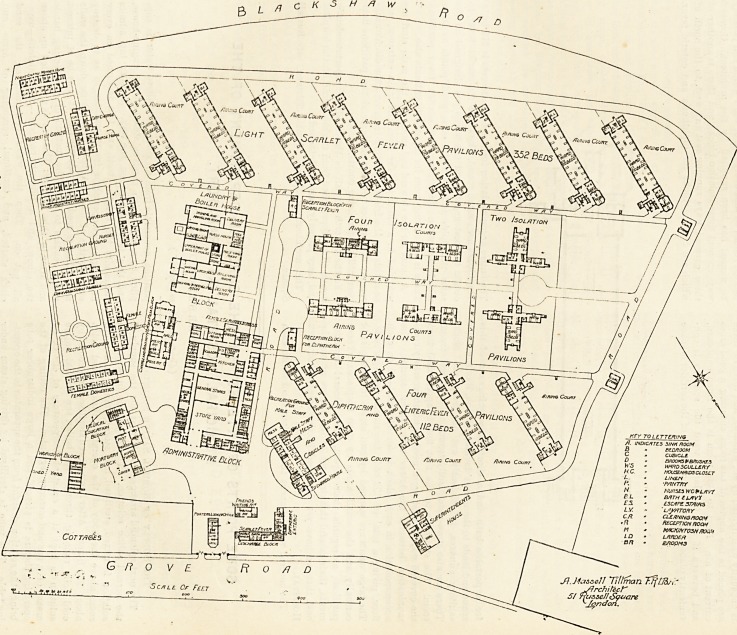


**Figure f2:**